# “I’d rather do that (Knee Control) than be injured and not able to play”: a qualitative study on youth floorball players’ and coaches’ perspectives of how to overcome barriers for injury prevention exercise programme use

**DOI:** 10.1136/bmjsem-2024-001953

**Published:** 2024-08-28

**Authors:** Ida Åkerlund, Sofi Sonesson, Hanna Lindblom, Eric Hagelin, Siw Carlfjord, Martin Hägglund

**Affiliations:** 1 Unit of Physiotherapy, Department of Health, Medicine and Caring Sciences, Linköping University, Linköping, Sweden; 2 Sport Without Injury ProgrammE (SWIPE), Department of Health, Medicine and Caring Sciences, Linköping University, Linköping, Sweden; 3 Unit of Public Health, Department of Health, Medicine and Caring Sciences, Linköping University, Linköping, Sweden

**Keywords:** Exercise, Adolescent, Implementation, Sports medicine, Prevention

## Abstract

This study explored youth floorball players’ and coaches’ perspectives on using the injury prevention exercise programmes (IPEPs) *Knee Control* or *Knee Control+* (*Knee Control* programmes) and how to overcome barriers to programme use. We used a qualitative design with eight semistructured focus group discussions, six with players, 11–17 years old (n =42) and two with coaches (n =12). Data analysis followed the principles of qualitative content analysis. Three main categories emerged: challenges related to preventive training, promoting factors and solutions to facilitate the use of preventive training. To overcome barriers, players and coaches gave examples of how to tailor preventive programmes, such as adding joyful sport specific components. Player-perceived improved performance, with increased strength and speed from the preventive training, could be a promoting factor to increase motivation and enable IPEP use. Players and coaches offered examples of how to adapt and progress the preventive training by progressing gradually and choosing exercises that fit the team. Coaches emphasised that preventive training is important but difficult to prioritise in time-limited training sessions. Coaches’ suggestions to overcome barriers were through collaboration and support from other coaches, to start using the IPEP at an early age, to keep it simple and motivating the players with, for example, positive role models. Players found the *Knee Control* exercises boring but necessary for injury prevention. Sometimes, coaches felt uncertain of their competence to use the *Knee Control* programmes and wished for support from the federation, club and other coaches. Players and coaches shared ideas on how to overcome barriers to IPEP use, such as to increase players’ motivation, having a good structure, setting up routines for preventive training and to tailor the preventive training to the team. These findings can be used to further develop practical workshops and recommendations for programme use for players and coaches in youth team ball sports.

**WHAT IS ALREADY KNOWN ON THIS TOPIC**

- Injury prevention exercise programmes (IPEPs) effectively prevent injuries in youth team ball sports. Successful implementation of these programmes is necessary to maximise programme effectiveness.

**WHAT THIS STUDY ADDS**

- Coaches expressed that support from other coaches, clubs, federations and further education could promote adoption and use of an IPEP.
- Players and coaches discussed similar solutions to overcome barriers to IPEP use, such as good structure, routines for preventive training and to tailor the preventive training to the team.
- Role models, creating a tolerant environment and providing information about the preventive effect were suggestions to increase the players’ motivation.

**HOW THIS STUDY MIGHT AFFECT RESEARCH, PRACTICE OR POLICY**

- These findings can further support the implementation of IPEPs in youth floorball by, for example, developing practical workshops and recommendations for programme use for players and coaches.

## Introduction

Floorball is a popular team ball sport in Northern Europe. It is a pivoting sport played indoors with a plastic stick and a light plastic ball. Floorball, like other team ball sports, is associated with the risk of injuries.^1 2^ Injury prevention exercise programmes (IPEPs) have been developed to mitigate this risk.^3^ IPEPs such as *FIFA 11*+, the *Knee Control* and *Knee Control+* (*Knee Control* programmes) have been efficacious in preventing injuries in structured randomised controlled trials in football,^4^,^6^ floorball^7^ and handball.^8^

Successful implementation of efficacious programmes, with widespread adoption, fidelity and maintained use, is necessary to achieve the full effect of IPEPs in sports.^9^ Although players are the intended end-beneficiaries of IPEPs, the decision to adopt a programme will often be the responsibility of team staff members such as coaches.^10^ However, a coach expressing a positive attitude towards IPEPs and an intention to start using them does not ensure subsequent use,^9 11 12^ as implementation is influenced by multiple factors characterised as either barriers or facilitators. Previous studies on youths and adults in various team ball sports have described several barriers (eg, low motivation among players and programme-specific factors, such as boring and time-consuming exercises) and facilitators (eg, belief in the IPEP’s injury preventive effects) for implementation and use of IPEPs.^13^,^21^ However, barriers and facilitators may be context specific (eg, floorball is an indoor sport, where teams have limited access to the court), which justifies studies in different sporting contexts and knowledge about how players and coaches can be supported in successful adoption and use of IPEPs is needed.^22^

There are some studies in youth team sports on the community level^1516 19 21 23^,^25^ and one previous survey study in youth floorball that described coaches’ barriers to and facilitators of *Knee Control* programme use.^20^ Based on these results, this qualitative study aimed to explore youth floorball players’ and coaches’ perspectives on using the *Knee Control* programmes and how to overcome barriers to programme use.

## Methods

### Study design and setting

We used an explorative qualitative design grounded in the constructivist paradigm, which studies multiple realities constructed by different groups of people.^26^ We used focus group discussions (FGDs) due to their interactive nature, which facilitates the expression of beliefs, attitudes and experiences providing a broad view of the subject.^27^ The study followed the consolidated criteria for reporting qualitative studies.^28^ The setting was community-level youth floorball in one regional district (Östergötland) in Sweden.

### Participants and recruitment

The participants were male and female youth floorball players aged 11–17 years and floorball coaches for players of the same ages who had experience using *Knee Control* or *Knee Control+* (table 1). First, we emailed one coach per team (in total 82 coaches) for all youth teams with players 11–17 years old in the district with study information. One week later, a purposeful sampling procedure was conducted,^29^ in which we strategically selected players and coaches through maximum variation sampling based on the sex, age group of players and geographic location. Two authors (IÅ and EH) contacted coaches over the telephone to provide additional information. They ensured the team had experience with *Knee Control* or *Knee Control+* use during the 2022/2023 study season. If so, the players and/or coaches were asked to participate in the study. However, the authors had no contact information for the players, therefore the coaches asked their players about preliminary interest to participate in the study. After this, interested players and their legal guardians received information about the study and an invitation to participate from the researchers via the coach. Therefore, there is no data on how many players declined participation in the study. Most respondents had not had any contact with the researchers before the FGDs. However, HL and a colleague in the research group have had a practical workshop on *Knee Control+* with players in one team that participated in the current study.

**Table 1:** Description of the injury prevention exercise programmes *Knee Control* and *Knee Control+*

Description of the programme	*Knee Control*	*Knee Control+*
Programme content	6 main exercises, 4–5 steps of progression, including partner exercises	Running warm-up and 6 main exercises, 10 steps of progression, including partner exercises
Dose	8–15 repetitions, 3 sets	30–60 s, 2 sets
Duration	10–15 min	15–20 min (including 5 min running warm-up)
Set-up	Part of the warm-up	Any time during the training session

### Data collection

Two semistructured interview guides (separate for players and coaches) were developed based on previous research on perceptions of *Knee Control* and barriers and facilitators for *Knee Control* use.^20^ They followed guidelines by Krueger and Casey^27^ (online supplemental material). The interview questions focused on experiences of using *Knee Control/Knee Control+*, perceptions of injury prevention, motivators and barriers to injury prevention training and how to overcome these barriers. Two pilot FGDs were conducted (one with players and one with coaches), but no adjustments to the interview guides were made, and data from the pilot FGDs were included in the analysis.

FGDs were held face-to-face and lasted between 28 and 34 min (total time 180 min) in the six player groups and 77–85 min (total time 162 min) in the two coach groups. A priori, based on the specific aim and strategic sampling, six player FGDs and two coach FGDs were deemed sufficient to reach information power according to Malterud *et al*.^30^ Coaches were believed to present more information-rich cases compared with the young players because they were older and could provide a stronger quality of the dialogue. The FGDs were conducted between March and May 2023. The player groups met in a room near the floorball hall before a training session. The coach groups met in a meeting room at the regional floorball federation. During FGDs, only the participants, the moderator and one assistant were present. Participants were encouraged to speak freely and share their experiences, and the discussions were characterised by a friendly atmosphere. Before the discussion, all participants answered background questions about their age and years of floorball experience, and coaches also answered a question about education in injury prevention training. The discussions were audio recorded and transcribed verbatim. The transcripts were not returned to the participants for comments or corrections.

### Data analyses

Data were analysed using qualitative content analysis to find patterns and core consistencies in the data.^26 31^ Transcripts were read several times to provide a comprehensive picture. One transcript was analysed and coded independently by three authors (IÅ, EH and SC) and discussed to achieve agreement on the coding strategy. After that, IÅ coded the remaining transcripts based on the coding strategy. Microsoft Excel was used to manage the data.

Codes were combined into subcategories emerging from the data, and subcategories with similar meaning were combined into generic categories. Generic categories with similar meaning were combined into main categories,^31^ all striving to be internally homogeneous and externally heterogeneous. All authors were involved in the analyses, and consensus discussions were held several times. IÅ read the transcripts during the analyses to ensure consistency between the data and the findings.

It was expected that some perspectives would be shared by players and coaches. Therefore, the inductively identified main, generic and subcategories from the player analysis were used deductively in the coach analyses. Additionally, new categories from the remaining coach data were added inductively.^31^

The data comprised 446 codes in the player analysis and 197 codes in the coach analysis. Participants were not invited to comment on the findings. Quotes capturing the essence of the FGDs were selected to illustrate the different generic categories.^27^ Selected quotes were translated into English and then retranslated into Swedish to preserve their meaning.

### Positionality

IÅ (female), a PhD student, was the moderator, and EH (male) participated as an assistant and took notes during the discussions. Both are physiotherapists and introduced themselves as such to the participants. Neither had any previous experience with FGDs. IÅ, HL, MH and SS are part of a research group with a prior understanding of floorball research. Senior authors (HL (female), SC (female), SS (female) and MH (male)) are all physiotherapists and PhDs with previous experience in qualitative studies, and one author (SC) is an implementation researcher with previous experience in FGDs.

### Patient and public involvement

The research question and the interview guides were inspired by floorball players’ and coaches’ responses to surveys about perceptions, facilitators and barriers of IPEP use in a previous study.^20^ Players or coaches did not take part in the planning or conduct of the study.

## Results

Players were recruited from 8 different teams into 6 FGDs, in total 42 players participated. A player FGD consisted of players of the same sex and from one or two teams from the same club (to create a safe and tolerant environment) (table 2). The players’ average floorball experience was 7 years (range 1–10 years), seven teams had experience with *Knee Control* and one with *Knee Control+*.

**Table 2:** Characteristics of the focus groups

Focus group	Male/female team	Players’ age range, years	Informants, n
Group 1, players	Female	13–15	8
Group 2, players	Female	12–13	8
Group 3*, players	Female	12–16	8
Group 4, players	Male	13–14	5
Group 5, players	Male	15–16	6
Group 6*, players	Male	11–13	7
Group 7, coaches	Male/female teams n=4/2	12–15	6
Group 8, coaches	Male/female teams n=3/3	11–17	6

* Focus group consisted of two different teams from the same club.

16 of the strategically selected coaches initially agreed to participate, but 4 declined participation just before the FGD, and thus 12 coaches from 12 teams participated in 2 FGDs. Four coaches represented teams that were included among the player FGDs. The coaches’ average age was 44 years (range 39–53 years), with an average floorball coaching experience of 8 years (range 3–25 years). All coaches had experience of either *Knee Control* (n=9) or *Knee Control+* (n=3). Most coaches (n=9) had no formal education in injury prevention training. One of the included coaches was employed by the regional floorball federation, the other coaches were non-profit parental coaches.

**Figure 1:**
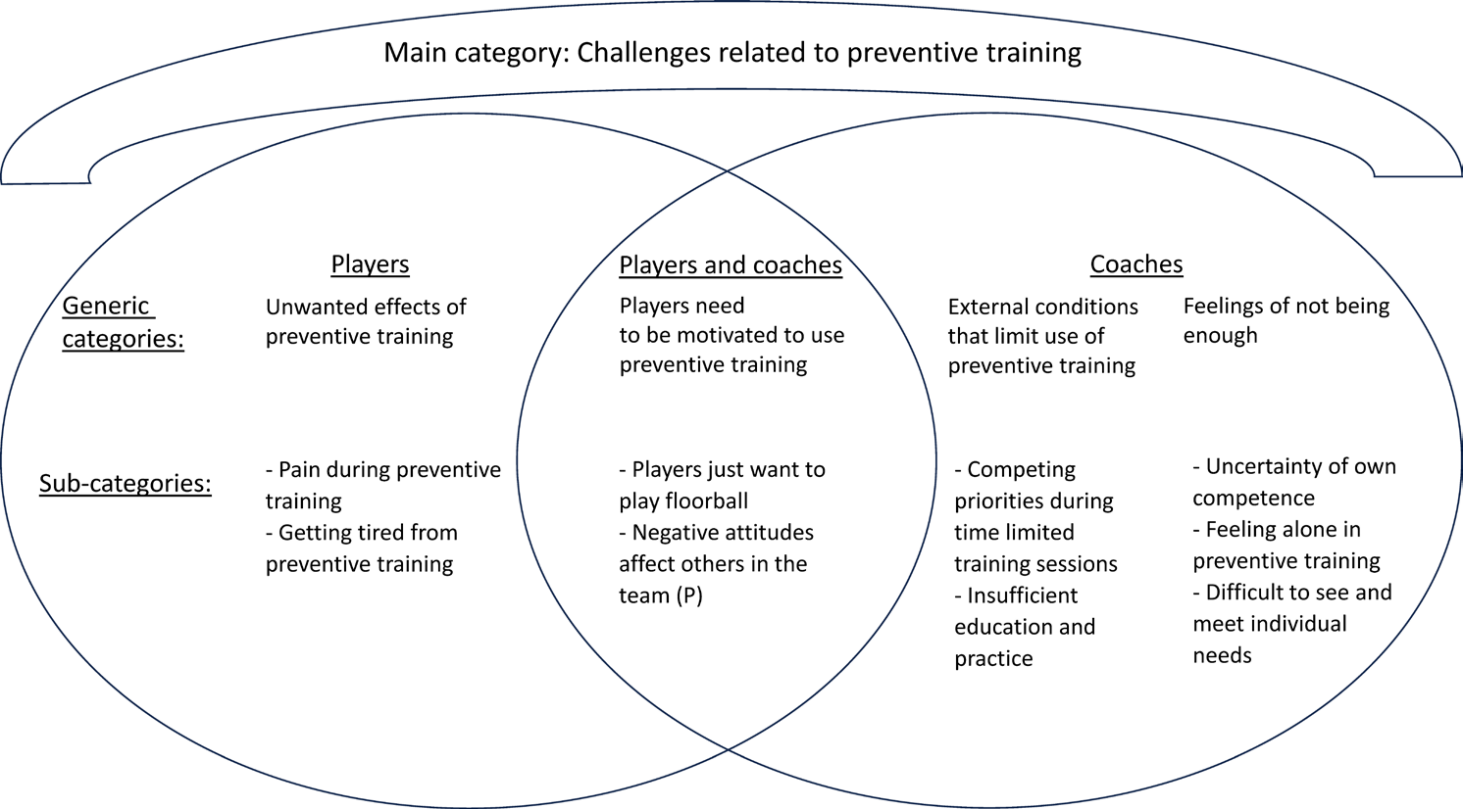
Generic and subcategories for players and coaches for the main category ‘Challenges related to preventive training’. (P): subcategory represented by players only.

The analysis resulted in 3 main categories shared by both players and coaches, 10 generic categories (3 player specific, 4 coach specific and 3 shared) and 27 subcategories (10 player specific, 11 coach specific and 6 shared) (figures1^3^). Illustrative quotes are used to exemplify the findings, (…) indicates omitted words.

**Figure 2:**
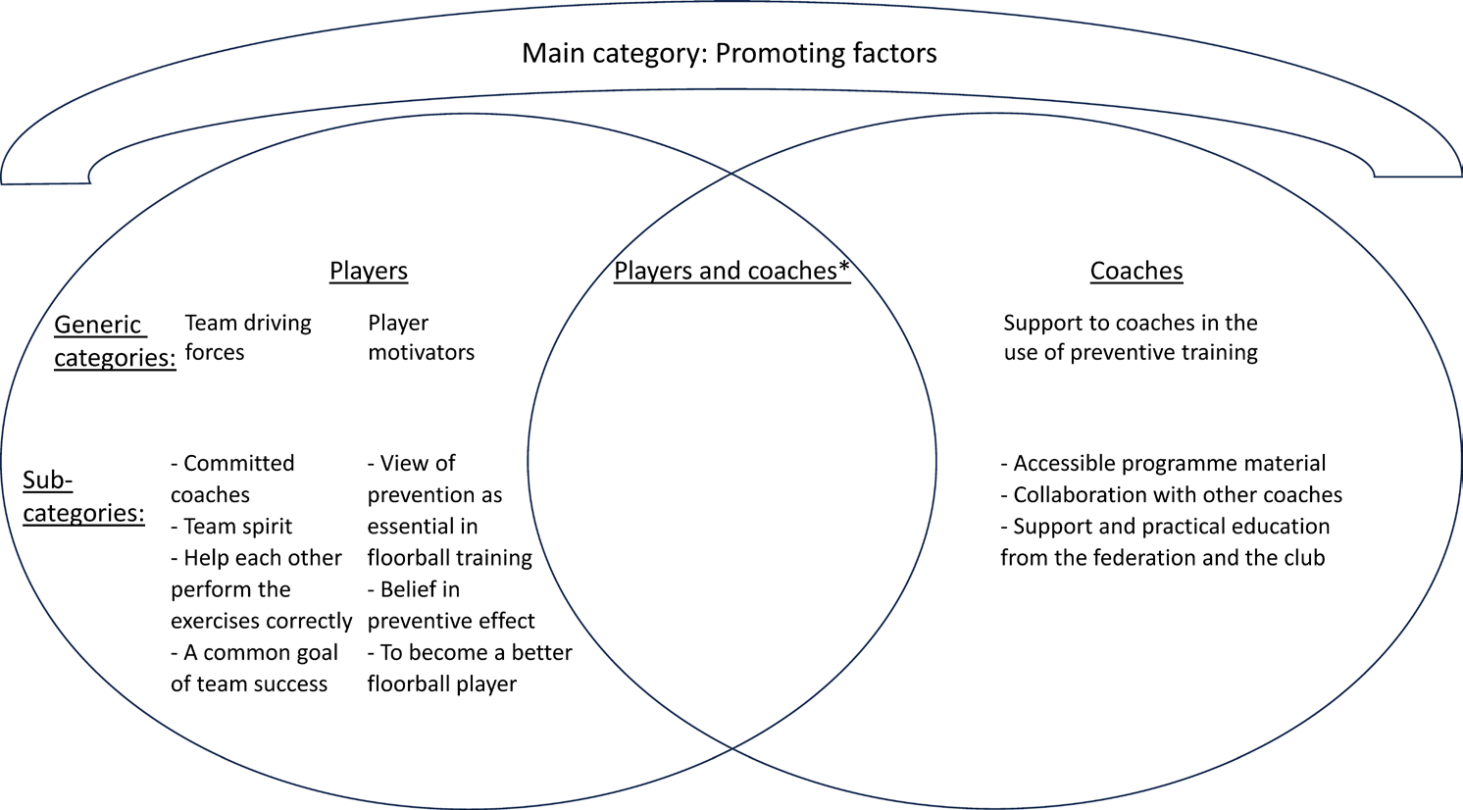
Generic and subcategories for players and coaches for the main category ‘Promoting factors’. *No shared generic or subcategories.

**Figure 3:**
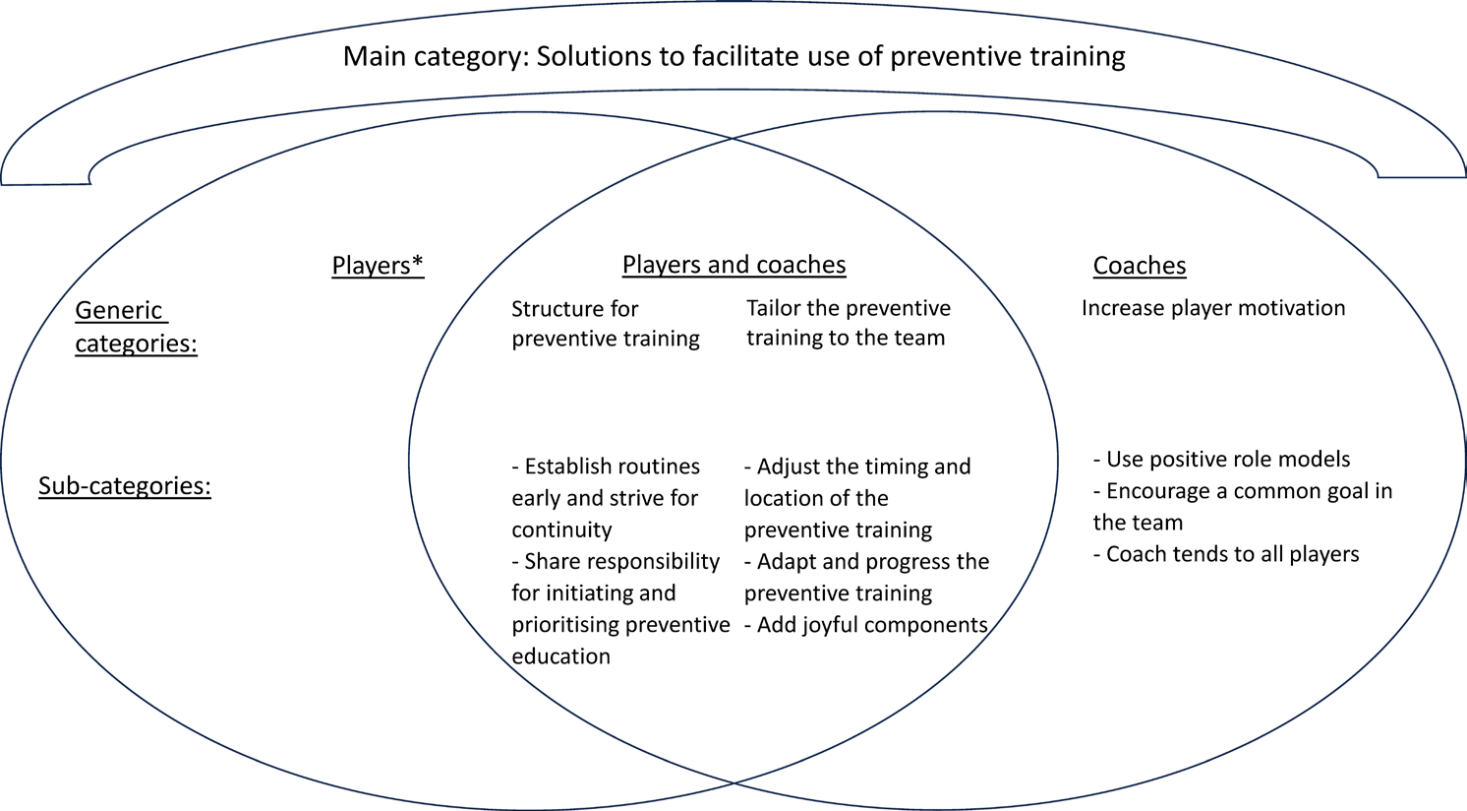
Generic and subcategories for players and coaches for the main category ‘Solutions to facilitate use of preventive training’. *No generic or subcategories for players.

### Challenges related to preventive training

This main category describes players’ and coaches’ challenges related to preventive training based on their perceptions of using *Knee Control* or *Knee Control+*. Players’ perceived physical challenges include pain and tiredness from the preventive training. Players and coaches agreed that players need to be motivated to use preventive training, and coaches described external and internal challenges that could limit the use of preventive training (figure 1).

#### Unwanted effects of preventive training

Players described unwanted effects, such as pain during preventive training, over-exertion and too intense preventive training. They related the pain to incorrect execution of the exercises or as being associated with a gradual onset injury.

“If you have your knee slightly over your foot, it hurts a lot.” (Group 6, player 1)

 “Sometimes you almost get a little too tired from the warm-up, I think.” (Group 5, player 5)

#### Players need motivation to use preventive training

Players and coaches expressed that players need more motivation to use preventive training. Players perceived the preventive exercises as boring and intruded on their floorball training time. Coaches expressed that players with the greatest need for preventive training were the least motivated. Coaches were also keen that players should think that floorball training is fun, and some coaches described that they skipped the preventive training if the players were not in the mood.

“You would rather play floorball than stand on one leg.” (Group 5, player 1)

 “I sometimes experience, sometimes when I do this type of training I become a boring coach, that I get tired if they don't do it properly. (…) It’s sometimes difficult to justify doing something that we know they won't find particularly fun.” (Group 7, coach 6)

Players described that peers in their team had different levels of ambition and motivation and that low motivation and bad attitudes could affect others negatively, both players and coaches. Evasions, such as tying a shoelace or going to the bathroom, were concocted to avoid preventive training. Players perceived that external factors such as long school days or fatigue from participation in other sports could negatively impact their motivation and attitudes about preventive training.

“If you are unmotivated and unfocused, then it can spread to other players so that they also start to make mistakes.” (Group 4, player 3)

#### External conditions that limit use of preventive training

Coaches described that preventive training was affected by limited access to the court and, therefore, had to prioritise which training components to include. Coaches described insufficient education about injuries and preventive training and requested practical workshops on how to use the programme correctly and how to perform the exercises from the club and federation.

“There is so much that needs to be included, agility needs to be included, and then when they get older, they should run a little longer so they get more fitness, there is so much besides floorball that you need to practice, what should you prioritise?” (Group 7, coach 2)

#### Feelings of not being enough

Coaches expressed that they felt uncertain of their competence and alone in prioritising preventive training with little support from other coaches in the team. Coaches experienced high demands that exceeded their perceived knowledge, and the responsibility of preventive training was a significant burden on them. Another challenge was to see all players in a heterogeneous team, to individualise the preventive training and meet many different needs, such as player interest and physical and mental maturity.

“It’s hard to get the coaching colleagues along sometimes because it’s like, “oh how good that you're here, then you take it (*Knee Control*).” You just, “okay”. So, you're sometimes quite alone with taking responsibility as well, because the others are more like “we play floorball, that’s why we're here”.” (Group 7, coach 2)

### Promoting factors

This main category describes team and individual promoting factors for players, and different types of support as promoting factors for coaches, which may contribute to overcoming barriers to implementation (figure 2).

#### Team driving forces

Players described committed coaches who offer support and feedback on exercise technique and stringent coaches who facilitate the use of preventive training as team driving forces promoting injury prevention. Other team driving forces included team spirit, whether players had good friendships within the team and whether they supported each other when they performed the preventive exercises.

“It is quite often that we encourage each other and especially the leaders are very good at encouraging. (…) You often want to get the best you can out of the training for the day.” (Group 5, player 6)

 “We also try to encourage and help each other if there is something that can be done better.” (Group 1, player 3)

#### Player motivators

Players believed that injuries are preventable and were driven by the knowledge that if an injury occurred, it could affect their ability to play floorball both now and in the future. Player-perceived improved performance with increased strength and speed from the preventive training can also be a promoting factor to increase motivation and enable IPEP use. Players mentioned that sharing a common floorball-related goal in the team helped them to find motivation for preventive training. Preventive training was considered an integral component of floorball training and a mandatory practice.

“You want to be able to play floorball for a long time, and to have the conditions to be able to do what you want to do without injury; something that you could have done something about.” (Group 2, player 5)

 “I mostly feel that it is good to avoid injuries. Injuries mean that you may be kept away from playing floorball that you like and then it’s better to do this (*Knee Control*) so that you last.” (Group 5, player 6)

#### Support to coaches in the use of preventive training

Coaches believed learning from and collaborating with other coaches in the same team, other floorball teams or coaches in other team ball sports was fruitful. Support and practical education from the club and federation were also desired to boost confidence. Coaches believed that accessible programme material could promote programme use, either as an application on a mobile phone with videos of the exercises or descriptions and photos of the exercises in a leaflet. The coaches also requested age-appropriate information about injuries and preventive training for the players.

“I also think that it is super important what kind of support you have in the team, coach colleagues, if you are a good team, you support each other. Of course, you have downs during a season when you are more or less motivated, but can you help each other and give each other some energy and tips and thoughts, etc. After all, we all contribute different parts.” (Group 7, coach 1)

### Solutions to facilitate use of preventive training

This main category describes players’ and coaches’ shared solutions to facilitate the use of preventive training—good structure and routines and tailoring the preventive training to the team—based on their experiences with *Knee Control* or *Knee Control+*. It also illustrates coaches’ solutions how to increase the players’ motivation to use preventive training (figure 3).

#### Structure for preventive training

Players and coaches agreed that having a good structure with established routines and continuity in preventive training is important. Coaches believed that establishing routines early on when players were still malleable was important. Some players agreed with the coaches that starting early in a player’s career was good, while others found that it could be difficult to have enough focus and motivation at a young age. Players and coaches acknowledged that the coach was responsible for preventive training, and coaches emphasised the need to prioritise prevention training to ensure its completion. Players and coaches agreed that it is important to involve the players in sharing responsibility for preventive training.

“I think like this, that you have a special time before the training, and that you have a routine and don't think every time, what are we going to do this time? Or now we just do this, and we do it every day before training.” (Group 3, player 2)

 “The girls have a solid grasp of this themselves, it’s just that you say now it’s warm-up and *Knee Control* and then they start. (…) Then they do their warm-up, and they do their jumps and their balance exercises and their squats and so on. It is really established among the players that this is what we do.” (Group 8, coach 4)

#### Tailoring of the preventive training to the team

Players and coaches discussed alternatives to tailor the preventive training to the team, such as to adjust the time when *Knee Control* is performed during training and the location to minimise the barrier of limited access to the court. Some players and coaches described that they had solved the issue of limited training time on the court by performing the preventive training outdoors or in a corridor next to the court before the court time started. Others described that they had longer training sessions and did preventive training on the court, sometimes at the end of the training.

Players and coaches gave examples of how to adapt and progress the preventive training to facilitate programme use; for example, performing the exercises for a set duration instead of counting repetitions, and players also described that they changed to another exercise to avoid pain or when they were unable to do an exercise due to injury. Players and coaches offered other examples of how to adapt and progress the preventive training by starting early, progressing gradually, keeping it simple, and choosing exercises that fit the team. Players and coaches wished to make the preventive training more fun by adding joyful components to facilitate programme use; for example, using a floorball stick and ball, adding competitive elements, and varying the exercises.

“If it works, you are outdoors, but in almost all training halls there is at least a small corridor where there is room for everyone. There are variations of these exercises where you don't need as much space. So, we are always warmed up when our training time starts and have finished these exercises and can continue with regular floorball when we get access to the court.” (Group 8, coach 2)

 “When we do partner exercises and things like that, you can compete a bit too and make it fun that way. It can certainly be boring, but you can make it fun too, by competing.” (Group 5, player 4)

 “I prefer the stick because it’s always with me and it’s associated with floorball. You have the stick for practice, and you have it for matches. So, we do squats with a stick, lunges with a stick, side bends, all that stuff.” (Group 7, coach 4)

#### How to increase players’ motivation

Coaches suggested that positive role models, such as older or elite players, could enhance players’ motivation and that role models can be used to create a positive culture surrounding preventive training. To encourage a common performance target, the coach could motivate the players by increasing team spirit and helping them understand why preventive training is important to become a better floorball player. Coaches suggested that they could participate in the preventive training together with the players, individualise the exercises, and create a safe and tolerant environment to address the challenge of seeing all players in the team.

“If you have watched a women’s match and watched the warm-up, (…) there are exercises and drills that the players pick up from there. (…) You inherit it, and it becomes like a culture.” (Group 8, coach 4)

## Discussion

The main new findings from this study were players’ and coaches’ solutions of how to facilitate use and overcome barriers to preventive training. Players and coaches agreed to focus on structure and tailoring preventive training and strategies to increase player motivation.

### Player and coach perceived challenges regarding programme use

Player-specific challenges in the present study included exhaustion from preventive training and the perception that the preventive training was too intense. This can be explained by an overall training load that is too high or by the fact that some players did not have the physical capacity to cope with the team’s preventive training and may have needed adapted training. While it would be preferable to tailor the IPEPs to the players’ needs,^32^ for practical reasons the team often uses the same exercises together. Other player-related challenges to IPEP use were reduced sport-specific training time and exercises that were perceived as boring and not sport-specific, which appear to be generic barriers in various youth team ball sports.^18 23 25^

Coach-perceived challenges to *Knee Control* use in the present study included competing priorities during time-limited training sessions and uncertainty of their competence, which have been reported earlier in various populations and IPEPs.^18 19 24 25 33^ In addition to these, coaches’ need for support from others in IPEP use are noteworthy challenges to overcome.

### Programme-related facilitators

The present study highlights the importance of incorporating players’ and coaches’ perspectives in IPEP implementation. The results illustrate shared (eg, to tailor the preventive training to the team) and separate perspectives (eg, supporting coaches in the use of preventive training) to facilitate IPEP use. Players and coaches described that they adapted the programme to their needs, aiming to make the training more joyful or to fit their players’ physical capacity. Player-initiated and coach-initiated adaptations may be positive regarding IPEP adoption and use, but there is currently no data on whether or not they affect the IPEP’s effectiveness. In line with the present results, some previous studies have proposed solutions to facilitate the use of preventive training related to programme-specific factors, such as having a flexible programme that is easy to individualise and adapt and the use of sport-specific equipment.^18 23 25^

### Promoting factors for programme use

Player-perceived promoting factors, such as having a positive team spirit, can promote the use of IPEPs when performing preventive training together as a team. Low player motivation for preventive training as a challenge perceived by players and coaches has also been described in other youth team sports.^18 19 21^ Players discussed different experiences to increase their motivation, like belief in the preventive effects of the IPEP, and experience of becoming a better floorball player. These are examples of intrinsic motivators that may facilitate behaviour change.^34^ According to the self-determination theory, basic psychological needs are autonomy, competence and relatedness.^34^ To increase players’ competence in injury prevention, they need information about floorball injuries and how to prevent them, theoretically and practically. Coaches proposed that this information can be relayed to players through written information and short videos with role models.

Coach-perceived promoting factors for IPEP adoption and use included to increase coaches’ knowledge and competence of the *Knee Control* programmes with support from clubs and federations, and collaboration with other coaches. Previous studies in youth team ball sports show that knowledge of the IPEP's preventive effects, continued education during the season and more training in the delivery of the IPEP can facilitate implementation.^18 19 21^ A previous study in youth floorball showed that high coach self-efficacy (belief in one’s ability to use the IPEP) was a promoting factor for the intention to maintain IPEP use.^20^

### Solutions to facilitate use of preventive training

As expected, players prefer sport-specific training to preventive training, but findings from the present study indicate that players understand the importance of preventive training. To enable the adoption of the IPEP, it is important to achieve acceptance and an attitude that preventive training is an essential part of floorball training.^17^ Players and coaches discussed practical examples of how to increase the adoption and use of the *Knee Control* programmes by prioritising preventive training in the team, establish routines early on, start simple with a few exercises, and progress gradually. A recent meta-analysis concluded that implementation workshops are valuable tools for successfully implementing IPEPs in youth team sports.^35^ Teams should start using an IPEP in the pre-season and continue performing the programme through the regular season to maximise its preventive effects.^36^ However, coaches may need ongoing support to maintain IPEP use.^37^

### Methodological considerations

The qualitative study design produces context-specific results. However, some identified barriers and facilitators are likely transferable to other team ball sports, such as the importance of player motivation, limited access to training courts, and limited training time, resulting in a need to prioritise. However, the implementation context is important to keep in mind when addressing barriers and facilitators to achieve maintained use of IPEPs.^38 39^ Participants in this study had experience with either of the *Knee Control* programmes, and their perceived challenges or solutions may not be transferable to other floorball players and coaches unfamiliar with the programmes.^40 41^

The total amount of data was considered sufficient to reach information power to answer the aim of the present study from players’ and coaches’ perspectives. Throughout the analyses, the authors had consensus discussions to improve the credibility of the findings. To ensure that all interpretations were in line with the data, the authors went back and forth from interviews to analysis, increasing trustworthiness.

A limitation of the study was that one coach worked at the floorball federation, which may have influenced the other coaches in the group to speak freely about the federation. Another potential limitation was that one author had a practical workshop with one of the teams. However, this author did not participate in the FGD, so the study’s trustworthiness was not considered to be affected.

A strength of this study was that it included players’ and coaches’ perspectives in the same qualitative analysis. This can be seen as a form of triangulation via data sources, where individual viewpoints and experiences can be verified against others. This provides the reader with a more comprehensive picture of the perspectives of two IPEP end-user groups and how these perspectives interact around challenges and solutions with IPEP use.^40^

## Conclusions

Players found the *Knee Control* exercises boring but necessary for injury prevention. Sometimes, coaches felt uncertain of their competence to use the *Knee Control* programmes and wished for support from the federation, club and other coaches. Players and coaches shared ideas on how to overcome barriers to IPEP use, such as to increase players’ motivation, having a good structure, setting up routines for preventive training and to tailor the preventive training to the team. These findings can be used to further develop practical workshops and recommendations for programme use for players and coaches in youth team ball sports.

## supplementary material

10.1136/bmjsem-2024-001953 online supplemental file 1

## Data Availability

The data generated and/or analysed during the current study are not publicly available due to their containing information that could compromise the privacy of research participants but are available from the corresponding author on reasonable request.
