# Supplementary material for: “I’d rather do that (Knee Control) than be injured and not able to play”: a qualitative study on youth floorball players’ and coaches’ perspectives of how to overcome barriers for injury prevention exercise programme use
Source: BMJ Open Sport Exerc Med. 2024 Aug 28;10(3):e001953. doi: 10.1136/bmjsem-2024-001953 (PMC11367341; doi:10.1136/bmjsem-2024-001953)
Supplement: online supplemental file 1 [file bmjsem-10-3-s001.pdf]

Supplemental material

Interview guide, players

Introduction

1. Can you tell us about injury prevention training in your team?

Transition

2. Why do you do injury prevention training?
   a. Motivation

3. What are your thoughts on injury prevention training?
   a. What is good/bad?
   b. What is fun/boring?
   c. How does your body feel during injury prevention training?

Key

4. Do you sometimes skip injury prevention training, and if so, why?

5. How can you increase the chance that the injury preventive training is performed?
   a. What could facilitate this? Players/coaches/other

6. How do you think your attitude as a player influences injury prevention training?
   a. How you perform the training
   b. What you say about the training
   c. Your body language and attitude

Ending

7. Is there anything else you would like to add?

Supplementary questions

- Do you want to explain more?
- Would you like to give an example?
- What do the rest of you think about this?

Interview guide, coaches

Introduction

1. Can you tell us about injury prevention training in your team?

Transition

2. Why do you do injury prevention training?
   a. Motivation

3. What are your thoughts on injury prevention training?

    a. What is good/bad?
    b. What is easy/hard?

Key

4. What challenges are there to implement and use injury prevention training?

5. What could be done about these challenges, is there anything that could make things easier?
    a. What could facilitate this? Coaches/players/the club/other

6. How do you see your role as a coach regarding injury prevention training?
    a. Responsibilities
    b. Knowledge, self-efficacy to carry out injury prevention training
    c. Attitude
    d. Commitment

7. How do you perceive the players' attitude towards injury prevention training?
    a. Does it affect injury prevention training?

Ending

8. Is there anything else you would like to add?

Supplementary questions

- Do you want to explain more?
- Would you like to give an example?
- What do the rest of you think about this?
